# Establishment and characterization of three canine ameloblastoma cell lines with genomic and transcriptomic profiles

**DOI:** 10.1371/journal.pone.0353197

**Published:** 2026-07-21

**Authors:** Stephanie Goldschmidt, Natalia Vapniarsky, John D. McPherson, Iris Rivas, Christine Ly, Abraham Morales, Daniel York, Robert Rebhun, Maria Soltero-Rivera

**Affiliations:** 1 Department of Surgical and Radiological Services, University of California, Davis School of Veterinary Medicine, Davis California, United States of America; 2 Department of Pathology, Microbiology, and Immunology, University of California, Davis School of Veterinary Medicine, Davis California, United States of America; 3 Department of Biochemistry and Molecular Medicine, University of California, Davis School of Medicine, Davis, California, United States of America; Nantes Université: Nantes Universite, FRANCE

## Abstract

Establishment of canine acanthomatous ameloblastoma (CAA) cell lines has profound importance in pre-clinical in vitro testing. The goal of this study was to develop, authenticate, and characterize three CAA cell lines. Cell lines were developed with standard cell culture techniques from dogs with naturally occurring CAA. Each cell line was authenticated as canine with a validated PCR panel. Epithelial cell origin was confirmed with pan-CK flow cytometry. Next generation DNA and RNA sequencing characterized features of the cell lines and compared them to the parent tumor. We confirmed that all cell lines maintained mutations seen in the parent tumors including a missense HRAS mutation and RTK-RAS pathway upregulation across all samples. Other common mutations (2/3 cell lines and their parent tumors) included *ADGRA3, DNAH7, F10, KIAA1671, OGFR, SLC6A17, SNX7, SPTBN5, TENM4*, and *YEATS2*. We compared the transcriptional profiles from our parent tumors and established cell lines to historical transcriptomic analysis of CAA and healthy gingiva. We confirmed the same canonical CAA molecular features of primary tumors across studies with distinct clustering from healthy gingiva. Further, we documented that the cell lines maintained upregulated genes that are seen within in vivo CAA across both studies including upregulated *GPC4* and *ETV5* as well as gene sets associated with the presence of epithelial-mesenchymal transformation, KRAS, Pi3K-AKT signaling, and hedgehog signaling pathways. Thorough characterization of the mutational and transcriptional profiles of the established cell lines, especially in context to how they differ from the parent tumors, sets the platform for translational in vitro testing.

## Introduction

Canine acanthomatous ameloblastoma (CAA) is the most common odontogenic tumor in dogs [1, 2]. Like other odontogenic tumors, it is classified as benign and carries a null (<1%) metastatic risk [3–5]. However, contrary to other odontogenic tumors, CAA has the propensity to be locally invasive requiring en-bloc surgical removal for long term remission. [6] Yet radical surgery, including maxillectomy and mandibulectomy, is associated with potential morbidity with one study reporting an up to 37% complication rate. [7] Alternative medical interventions, such as targeted chemotherapy or electrochemotherapy could help alleviate the morbidity associated with en-bloc surgical procedures and should be explored. Further, it has been demonstrated that canine ameloblastoma is an appropriate spontaneous model for human ameloblastoma [8–10], thus canine cell lines are an excellent first step in developing translatable interventions to improve treatment options for ameloblastoma across species.

To the authors knowledge there are no commercially available CAA cell lines for purchase and only one publication describing a conditional reprograming protocol to establish a CAA cell culture [8]. Although conditional reprogramming techniques allow the cells to better maintain their original karyotype and heterogeneity among numerous passages compared to immortalized cell lines; there is concern that lack of stromal cell population/tumor associated fibroblasts may affect their response to treatments [11]. Further, in the referenced publication the cell lines were not validated as canine, nor were the features of the cell lines fully characterized.

The aim of this study was to develop, authenticate, and characterize three CAA cell lines to allow for in vitro pre-clinical testing. Given the risk that cell line genetic material can alter from the parent tumor [12], we have provided in depth analysis of both the CAA cell lines and their parent tumor to effectively describe the cell line genomic and transcriptomic landscape and add to the existing studies which have included whole exome sequencing [8] and RNA seq [9, 10] of CAA primary tumors.

## Materials and methods

### Primary tumors

**Fig 1:**
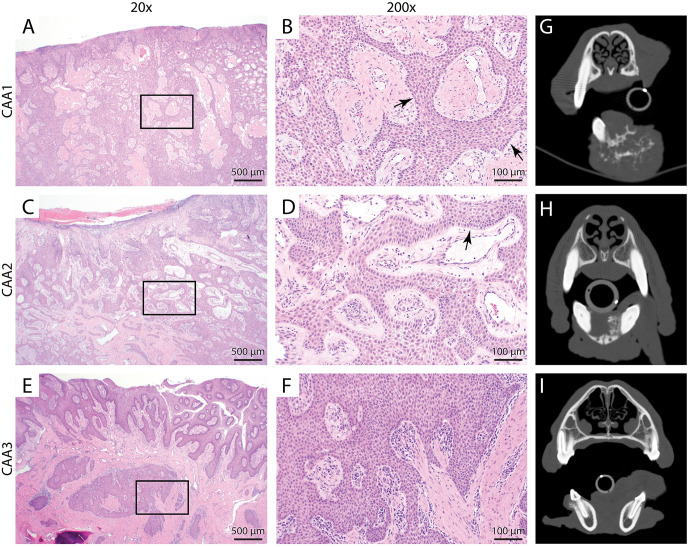
The Original Histology and Computed Tomography (CT) of the Canine Acanthomatous Ameloblastomas (CAA). A-B. Low and high magnification of the CAA # 1. The area enclosed in the black rectangle in A is shown at higher magnification in B. Note the palisading of the epithelial cells with anti-basilar nuclear positioning of epithelial cells (back arrows), which is indicative of an odontogenic origin, in addition to the sheet-like arrangement of the epithelial cells with a prominent, clear intercellular border (acanthomatous component). C-D. Low and high magnification of the CAA # 2. The area enclosed in the black rectangle in C is shown at higher magnification in D. Note the palisading of the epithelial cells with anti-basilar nuclear positioning of epithelial cells (black arrow), which is indicative of an odontogenic origin, in addition to the sheet-like arrangement of the epithelial cells with a prominent, clear intercellular border (acanthomatous component). E-F. Low and high magnification of the CAA # 3. The area enclosed in the black rectangle in E is shown at higher magnification in F. Note the sheet-like arrangement of the epithelial cells with a prominent, clear intercellular border (acanthomatous component). Low magnification photomicrographs, Bar = 500µm; High magnification photomicrographs, Bar = 100µm, All photomicrographs are H&E. G-I. Corresponding CT scans shown in the transverse plane from the clinical patients for CAA 1-3.

Tumor samples were harvested from four treatment-naïve clinical patients (Table 1) that presented to the UC- Davis Veterinary Medicine Teaching Hospital (Davis CA; IACUC protocol #22738, client consent to utilize patient tissue samples was obtained on the hospital intake form). Tumors were confirmed as ameloblastoma based on diagnostic imaging and histopathologic features (Fig 1). All tumors were reviewed by a single board-certified pathologist (NVA). One tumor failed to progress past the second passage and was discarded. This tumor had a very high keratin and amyloid component with a final pathology diagnosis following surgical excision of amyloid producing ameloblastoma.

**Table 1:** Ameloblastoma Cell Line and Primary Tumor (parent tumor) Characteristics.

Primary Tumor	Patient Signalment	Tumor location	Final Passage	Days of Culture	Cell line identifier
74-88-98	12 yo FS Jack Russel Terrier	Rostral Mandible	P8	59	CAA 1
74-01-66	12 yo MN Catahoula hound dog Mix	Rostral Mandible	P8	60	CAA 2
41-60-65	9 yo MN Pit Bull Terrier	Caudal Mandible	P7	58	CAA 3
N/A	7 yo FS Siberian Husky	Caudal Maxilla	P2* discarded	73	N/A

FS: female spayed, MN: male neutered, yo: year old, CAA: canine acanthamatous ameloblastoma

### Cell culture

Primary tumor samples were aseptically collected immediately following surgical excision and transported to the laboratory on ice. Collected samples (approximately 1–2 grams each) were bisected, with one section flash frozen for later sequencing and the other suspended in a 15 mL Corning® Dulbecco’s Modified Eagle’s Medium (DMEM) (Corning, Manassas, VA) supplemented with 1% penicillin-streptomycin-fungizone (Gibco, Grand Island, NY) for mechanical dissociation. In the biosafety cabinet under sterile conditions, the tumor sample was washed three times and mechanically dissociated with a razor knife into 1–2 mm pieces. The dissociated tissue was transferred into Corning® DMEM supplemented with 10% GenClone 25-514H GenClone™ Fetal Bovine Serum (FBS) (Genessee Scientific, Morrisville, NC), 1% penicillin-streptomycin-fungizone (Gibco, Grand Island, NY), 1% Modified Eagle’s Medium (MEM) Vitamin Solution (Gibco, Grand Island, NY), and 1% Modified Eagle’s Medium Non-Essential Amino Acids (MEM NEAA) (Gibco, Grand Island, NY) on 10 cm cell culture dishes (Fisher Scientific, Waltham, MA) at 37°C and 5% CO2. No genetic modifications were introduced to the cells to promote immortalization due to rapid and continued growth without intervention.

Once the initial plated cells with the dissociated tissue pieces adhered and reached 90% confluency, the cells were lifted with 0.25% trypsin-EDTA (Gibco, Grand Island, NY) and the cell suspension was put through a 40−100 µm cell strainer to remove any remaining tissue debris and be left with a single cell suspension. The cells were then counted and 1 x 10^6^ cells were plated to establish the first passage. At 90%−100% confluency the cells were passaged. At each passage, the cell monolayer and associated fibrobroblasts were lifted using 0.25% trypsin-EDTA and cell counts were obtained. During the initial growth phase, fibroblasts were diluted from the cell monolayer with repeated differential trypsinization. The population doubling times were calculated using the following formula: −0.693 (hours of growth)/ln (number of cells initially seeded/number of cells after growth) as previously described [13]. All cell lines were expanded to at least passage 7. Data from the total passages were averaged to calculate the doubling time for each cell line.

### Authentication of cell lines

Cell lines were submitted to IDEXX BioAnalytics (Columbia, MO) for short tandem repeat (STR) profiling and multiplex PCR (CellCheck™ Canine) to confirm species origin and absence of mycoplasma contamination. To verify epithelial origin, pan-cytokeratin (pan-CK) expression was assessed by flow cytometry (BD Biosciences, Milpitas, CA).

Cells were seeded in T75 flasks at a density of 2 × 10^6^ cells in DMEM and cultured for 4 days to reach confluency. Cells were then washed with 1 × phosphate-buffered saline (PBS), detached using 0.25% trypsin-EDTA, and neutralized with an equal volume of DMEM containing 10% fetal bovine serum (FBS). Following centrifugation at 400 × g for 5 minutes, cells were counted and 1 × 10^6^ cells were aliquoted into flow cytometry tubes in 400 µL of flow cytometry buffer (10% equine serum, 100 mM EDTA, 0.01% NaN_3_).

For viability controls, one aliquot was maintained as live cells, while a second aliquot was heat-treated at 65°C for 1 hour to generate dead cells; these were subsequently combined to create a mixed live/dead control. Dead cells were excluded using Fixable Viability Dye eFluor™ 780 (eBioscience, cat. no. 65-0865-14).

Prior to antibody staining, cells were permeabilized using permeabilization buffer (BD cytopem Cat. No. 554714, BD biosciences) based on preliminary validation demonstrating that permeabilization is required for pan-CK detection. Anti-pan cytokeratin AF488 antibody (Invitrogen, Waltham, MA) was applied at a 1:50 dilution and incubated for 30 minutes at room temperature in the dark. Canine hepatocytes derived from a necropsy specimen were used as positive controls for antibody titration and validation. Following incubation, cells were washed twice with flow cytometry buffer.

Data acquisition and analysis followed a sequential gating strategy: debris was excluded based on forward and side scatter, followed by live/dead discrimination using the viability control on the FL-4 channel. Pan-CK expression was then quantified as the percentage of FITC-positive cells within the live cell population using the FL-1 channel.

### Next generation sequencing

Genomic DNA was isolated from each cell line and primary tumor using the DNeasy Blood and Tissue Kit (Qiagen, Valenica CA USA) using the optional RNase step according to the manufacturer’s standard protocol. DNA concentration and purity were assessed using a NanoDrop 2000 Spectrophotometer and Qubit fluorometer (both ThermoFisher Scientific, Waltham, MA, USA).

Genomic DNA was prepared for sequencing using the Library Preparation EF 2.0 with Enzymatic Fragmentation and Twist Universal Adapter System according to manufacturer protocols (Twist Bioscience). Indexed, whole genome sequencing (WGS) libraries were pooled and multiplex sequenced on an Illumina NovaSeqX Plus System (150-bp, paired-end) to reach over 2X genome coverage. Target enrichment for whole exome sequencing (WES) libraries was performed using the Twist Alliance Canine Exome panel (40.5 Mb target region, > 17,000 genes), and sequencing was done on the Illumina NovaSeqX plus platform to achieve > 100X coverage (150-bp, paired-end).

Sequence reads were trimmed and aligned using canFam6 canine genome (UCSC Genome Browser assembly ID: canFam6;Dog Genome Sequencing Consortium Dog10K_Boxer_Tasha Oct. 2020; Accession ID: GCF_000002285.5) combined with chromosome Y obtained from canFam3.1 (UCSC Genome Browser assembly ID: canFam3; Broad Institute CanFam3.1 Sep. 2011 Accession ID: GCA_000002285.2) utilizing an Illumina DRAGEN (Dynamic Read Analysis for Genomics) Ultra-Rapid Next Generation Sequencing Data Analysis Platform. [13]. Q30 bases were greater than 92%, with median genome coverage of 89- to 95-fold across the 3 genomes. The DRAGEN was also used to generate variant VCF files for further analysis.

RNA was isolated from flash frozen tumor resections using Trizol reagent (ThermoFisher Scientific, Waltham, MA, USA) following manufacturer’s instructions and further purified using the RNeasy kit (Qiagen Valenica CA USA) with the optional on-column DNase step according to the manufacturer’s protocols. Total RNA was eluted from the columns in nuclease-free water and stored at −80°C. RNA concentration and purity were assessed with a NanoDrop 2000 Spectrophotometer (ThermoFisher Scientific, Waltham, MA, USA) and quality assessments (e.g., RNA integrity) were made using an Agilent 2100 Bioanalyzer (Agilent Technologies, Santa Clara CA USA).

Indexed, stranded mRNA-seq libraries were prepared from total RNA (1000 ng) using the KAPA Stranded mRNA-Seq Kit (Roche, Indianapolis IN USA) according to the manufacturer’s standard protocol for mRNA capture, fragmentation, random-primed first strand synthesis, second strand synthesis with dUTP marking, A-tailing, adaptor ligation, and library amplification. Libraries were pooled and multiplex sequenced on an Illumina NovaSeqX Plus System (150-bp, paired-end, > 30 × 10^6 reads per sample).

### Bionformatics pipeline

WES VCF data were filtered using snpEff and snpSift [14, 15] for quality, depth of coverage, removal of known breed single nucleotide polymorphisms (SNP) [16–18], normal SNP from an in-house collection of canine samples, black-listed SNP occurring in repeat-masked regions and due to observed frequent misalignments and functional impact. Variants were visualized using R package maftools. [19]

Low-pass WGS aligned reads were analyzed using R packages Ace, BSGenome and QDNASeq [20–22]. A custom BSGenome object was created for use with genome canFam6.

RNA-seq data were analyzed using R packages DESeq2 for differential expression between groups [23] and fgsea, clusterProfiler for pathway analyses [24, 25].

R (ver 4.5.1; [26], R-Studio (ver 2025.05.1 + 513; [27]) and other R packages used include ggplot2, dplyr, tidyverse, pheatmap, ggrepel, ComplexHeatmap, vcfr, biomart, gage, topGO, RNAseqQC,clusterprofiler ConsensusClusterPlus [28–41]. Variance stabilizing transformation (VST) was used for data transformation (DESeq2 vst function) with PCA generation using DESeq2 plotPCA (top 500 most variable genes; Singular Value Decomposition (SVD) using stats::prcomp).

## Results

**Fig 2:**
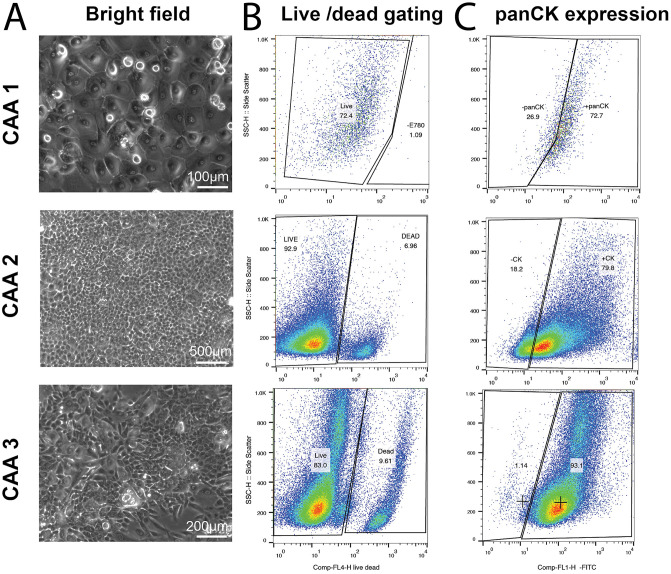
Histologic Morphology and PanCK Flow cytometry of Established Cell lines. A. Cell morphology of the three cell lines (CAA 1, CAA2, and CAA3, respectively) showing mostly uniformly stellate cells and no fibroblasts at passage 7−8. At various magnifications, bright filed microscopy, Bars 100µm, 500µm, and 200µm. B. Flow cytometry of the three cell lines showing live dead discrimination gating step, with CAA1, 2, and 3 cells lines exhibiting viability of 72.4%, 92.9%, and 83%, respectively. C. Flow cytometry of the three cell lines demonstrating panCK immunoreactivity, with CAA 1, 2, and 3 having 72.7%, 79.8%, and 93.1% immunopositivity for panCK, respectively. SSC (side scatter) is on the Y axis, while Fluorescence channels FL4 (live/dead) and FL-1 (FITC) are on the X-axis, in B and C columns, respectively.

Three validated canine cell lines were successfully established using traditional cell culture techniques. The average in vitro doubling time was 19 hours. The cell culture morphology was mostly uniformly stellate, with moderate anisocytosis and anisokaryosis. with occasional very large cells and rare multinucleated cells. All cell lines were confirmed to have strong pan-CK immunoreactivity with flow cytometry (Fig 2).

### Genetic alterations

There was no chromosomal loss or gain in CAA-1. CAA-2 had a subclone (approximately 70% of cells) with a chromosome 13 and 29 gain, which was not present in the primary tumor. However, the primary tumor did have a subclone (approximately 20% of cells) with a chromosome 27 deletion. CAA 3 had a subclone (approximately 80% of cells) with a gain of chromosome 18, which was not present on the primary tumor (S1 Fig).

**Fig 3:**
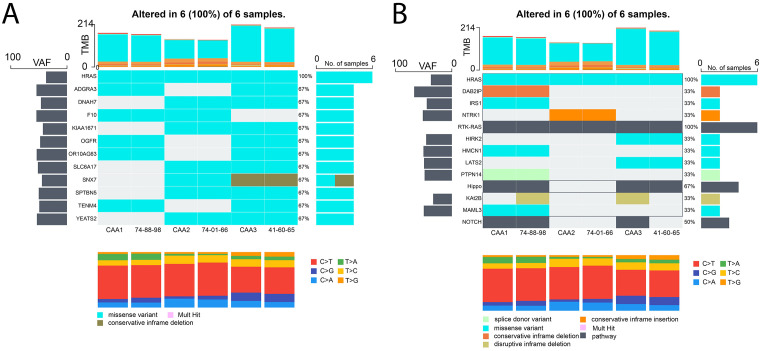
DNA Mutations Detected on Whole Exome Sequencing of the Cell Lines and Parent Tumors. Variant Allele frequency (VAF) is shown and presence of the mutation in 0-6 of the total samples. The type of mutation type is denoted by color. The most repeatable mutations that are present within 2/3 cell lines and parent tumors are shown on the left panel. Other mutations seen in only a subset of cell lines and/or parent tumors, as well as common pathway alterations are included in the right panel.

The most common mutation, which was found in all three cell lines and their parent tumors was a missense variant mutation of *HRAS* (p.Gln61Arg) with associated upregulation of the RTK-RAS pathway. Two out of three cell lines and their parent tumors also had missense mutations in *ADGRA3, DNAH7, F10, KIAA1671, OFGR, OR10AG63, SLC6A17, SPTBN5, TENM4*, and *YEATS2* (Summarized in S1 Table). Other alterations were not shared among 2/3 cell lines (either not present or different type of genetic alterations in the different cell lines) or were dissimilar between the parent tumor and the cell line (Fig 3). There were no significant co-occurrences of any mutations. (S2 Fig).

### Differential gene expression analysis

**Fig 4:**
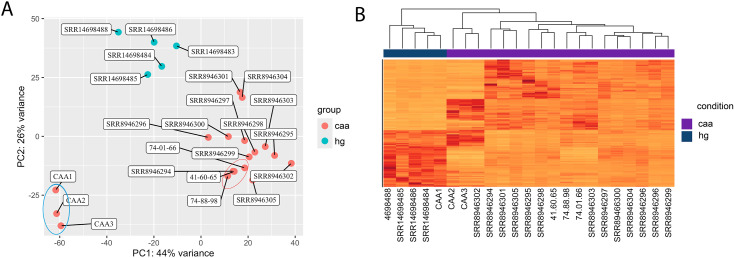
Principal Component Analysis and Clustering Heat Map Visually Depicting the Differentially Expressed Genes Between the Primary Tumors, Established Cell Lines, and the Historical CAA and Healthy Gingiva (hg) samples. A: Primary component analysis showing our primary tumors (red circle) and established cell lines (blue circle) compared to published CAA and hg samples. B) Clustering heat map depiction of differentially expressed genes in the cell lines (CAA1-3) and the primary tumors (purple) compared to hg (blue) again showing shared genetic expression in the cell lines, which was distinctly different from the primary tumors, and the healthy gingiva.

The next step in analysis was to determine the gene expression alterations occurring in the cell lines compared to the parent tumors. To assess whether the parental tumors from which the cell lines were derived recapitulate canonical CAA molecular features, we compared our data with published CAA genetic signatures and unmatched normal tissues [10]. Subsequent hierarchical clustering of the differential expressed genes (DEGs) and heatmap visualization demonstrated clustering of our parent tumors with historical CAA tumor data. PCA of variance-stabilized RNA-seq count data demonstrated clear separation between sample groups along principal component 1 (PC1), which captured the greatest proportion of transcriptional variance across all samples (Fig 4A). The published CAA tumor samples and our primary tumors clustered together, confirming that our parent tumors recapitulate canonical CAA transcriptional features. Notably, the three established cell lines formed a distinct cluster, separating from both the tumor samples and the healthy gingiva along PC1. Hierarchical clustering of differentially expressed genes (DEGs) identified between tumor and healthy gingiva further demonstrated clustering of parent tumors with historical CAA data, with the cell lines occupying an intermediate position between tumor and healthy gingiva (Fig 4B). It is important to note that this clustering analysis was performed on genes pre-selected for differential expression between tumor and normal tissue, which introduces a directional bias in the positioning of the cell lines; this is acknowledged as an analytical limitation.

**Fig 5:**
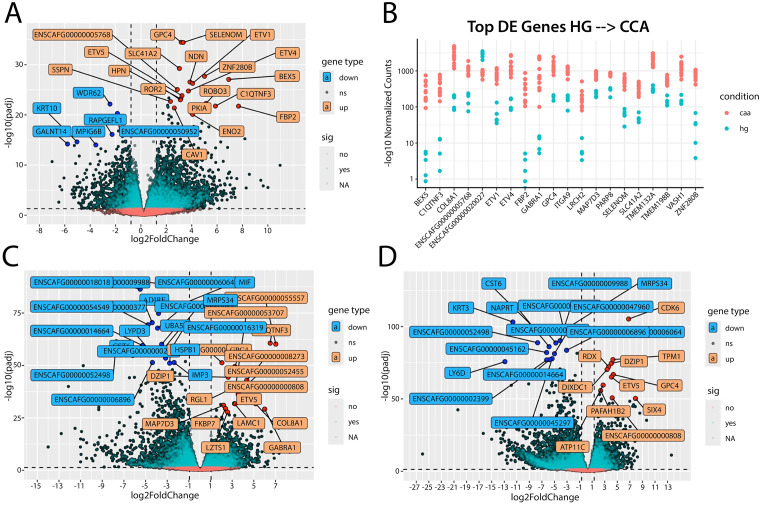
Differentially Expressed Genes Between Primary CAA, Established Cell lines, and Healthy Gingiva (hg). A: Volcano plot depicting differentially expressed genes between all CAA (our primary tumors and historical data) compared to historical controls (hg). B: Top differentially expressed genes between CAA (Our primary tumors and historical data) and hg. C: Volcano plot depicting the differentially expressed genes between primary CAA tumors (parent) from this data set only compared to historical controls (hg). D: Volcano plot depicting the differentially expressed genes between the cell lines compared to historical controls (hg).

The most upregulated genes separating CAA from healthy gingiva included *C1QTNF3, BEX5, COL8A1*, while most downregulated genes include *WDR62, RAPGELF1*, and novel lncRNA *ENSCAFG00000050952* (Fig 5A-B). Evaluation of the differentially expressed genes between the primary CAA from only this data set confirmed the same canonical changes as historical data (Fig 5C). The established cell lines had a different transcriptional profile compared to the primary CAA tumors yet maintained upregulation in *ETV5* and *GPC4* compared to healthy gingiva (Fig 5D).

### Pathway analysis

**Fig 6:**
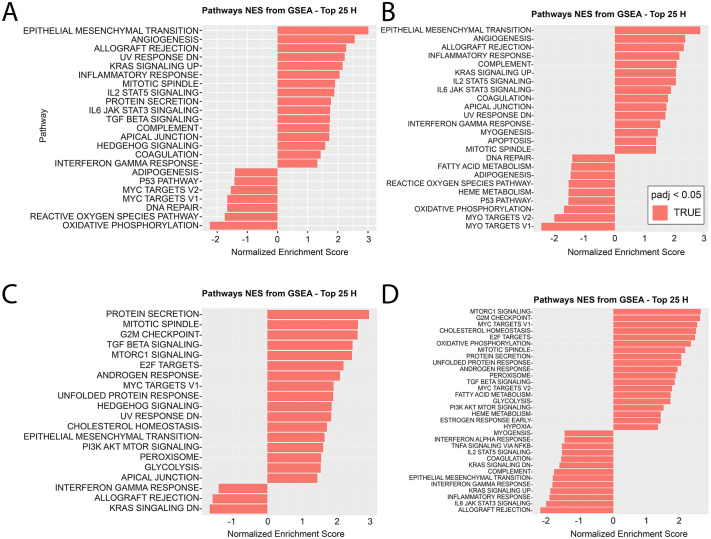
Functional Analysis of Primary CAA, Established Cell lines, and Healthy Gingiva (hg). Gene set enrichment analysis was performed using the MSigDB Hallmark gene signature collection. Bar plots depict the top 25 gene sets exhibiting statistical differential enrichment or depletion for the comparisons of A) All primary CAA (historical data and our data) to historical controls (hg) B) parent CAA (this data only) to historical controls (hg) and C) established cell lines to historical controls (hg). D) established cell lines compared to parent tumors.

Gene Set Enrichment Analysis (GSEA) was performed to gain insight into the functional implications of the transcriptional alterations that were identified by comparative analyses of the primary tumors, cell lines, and healthy gingiva. Notable enriched gene sets in primary CAA compared to healthy gingiva included epithelial mesenchymal transformation (EMT), angiogenesis, inflammatory response, KRAS, and the IL-6-JAK-STAT pathway. The same enriched pathways are retained when comparing all CAA (our data and historical data) to controls versus solely our parent CAA tumors to controls. A clinically notable different pathway that was only present in our set (both parent and cell lines) is upregulation of hedgehog signaling (Fig 6). When comparing the cell lines to healthy gingiva, clinically impactful pathways that were maintained included EMT and hedgehog signaling.

## Discussion

This is the first paper to describe the successful establishment of ameloblastoma cell lines in dogs. Cell culture was successful in three of four collected tumors following traditional culture techniques. Of interest, the tumor that was unable to sustain ex-vivo growth was an amyloid producing ameloblastoma. The presence of amyloid likely altered growth conditions of the cells, and for future cell work, it is recommended amyloid producing ameloblastomas are avoided. The CAA culture progressed rapidly and maintained epithelial morphology of the parent tumor, similar to what is described in humans. [42]

The cell lines were confirmed as canine with validated techniques. To further verify their lineage as epithelial, PanCK flow cytometry was performed. To the authors knowledge, this is the first report describing the use flow cytometry PanCK immunolabeling to confirm a canine cell line as epithelial in origin. This technique has been used in human bronchial epithelial cells with good success [43] but has never been evaluated in the context of oral cavity tissue. A limitation of this technique is that oral squamous cell carcinoma is also expected to be panCK positive. [44] Thus, although this technique confirms the cell line is epithelial, it does not clearly differentiate ameloblastoma from other epithelial oral tumors. Yet, the DNA and RNA analysis found driving mutations and transcriptional signatures that have been frequently documented in ameloblastoma [8–10], supporting their odontogenic origin.

Specifically, whole exome sequencing revealed all cell lines had an *HRAS* mutation which was also present in the parent tumor as well as aberrant RAS-RTK pathway activity. *HRAS* has been well documented as a driver mutation in CAA and human ameloblastoma through aberrant RAS activity. [8–10]. Maintenance of this aberration in our cell lines position them to be a useful tool for in vitro canine and translation human testing.

Other mutations that were found in 2/3 cell lines and their parent tumors included *ADGRA3, DNAH7, KIAA167, SPTBN5*, and *TENM4*, which have all been implicated with various cancer types and been shown to have tumorigenic activity with overall low cancer specificity [45–48]. None of these mutations have been directly implicated in oncogenesis of human or canine ameloblastoma, and their potential role in ameloblastoma development and/or progression is largely unknown and should be further investigated.

RNA-seq revealed that the parental tumors from which the cell lines were derived recapitulated canonical CAA molecular features. There was clear PCA clustering and structured hierarchal heat map visualization between our parent CAA tissue and historical data. Accordingly, there were shared up and down regulated genes within both data sets. Further, pathway analysis confirmed enrichment in similar pathways included EMT, angiogenesis, and KRAS. Interestingly, our parent tumors also had upregulation in the hedgehog pathway. This has been previously documented in human ameloblastoma, although it is normally mediated by *SMO* mutation [49, 50], which was documented in our set.

Unsurprisingly, our cell lines had different transcriptional signatures from the parent tumor and clustered separately on PCA and hierarchal heat map analysis. This is expected, because to maintain themselves in the cell culture settings, neoplastic cells must shift and adjust to in vitro growth within a plastic container. With time the cell lines will also become more homogenous shifting away from the heterogenous tumor population. Furthermore, in the absence of immune surveillance and the need to evade immune attack by the host, cultured neoplastic cells may abandon expression of genes needed for immune evasion in vivo. Interestingly, when comparing the DEGS between the cell lines, parent tumors, and the healthy gingiva, it can be seen that the cell lines are as dissimilar to healthy gingiva as they are to the parent tumors (Fig 4b, separate clustering from both). This likely is a direct reflection that the cell lines become more homogenous and lose the need for immune evasion and structural invasion in cell culture compared to in vivo, which are also not genetic modifications required in health. How these cell lines would act in a xenograft model and if they would recapitulate the tissue invasion and structural histology of ameloblastoma would have been an interesting addition to this study to further classify the research potential of these cell lines.

Further, it is important to note that we utilized low passage cell lines for our analysis, which may still contain a mixed cell population that may have potentially skewed the molecular findings. Although there is a scientific merit for immortalized cancerous cell lines, regarding their benefits in providing indefinite source of biological material, and reduction of donor-to-donor convenience [51,52], their practical use for development of curative therapies is rather limited [53]. Specifically, numerous studies in humans have reported that parent tumors and cell lines are genetically distinct [54–58], and the degree of genetic aberrance depends largely on the cell line with some being more dissimilar than others, but most clustering separately from the parent tumor on principle component analysis. Large scale studies have worked to compare commercially available cell lines and the tumor genome atlas to help guide the choice of cell line based on preservation of the most impactful and recurrent genetic alterations and gene expression patterns, as many commonly used cell lines represent outliers from the parent tumors [54–58]. Accordingly, careful choice of cell lines that maintain the genetic alteration of interest, and use of low passage cell lines that are less prone to genetic drift [53] is recommended. Our cell lines maintained the most important driver mutations and pathway upregulations, including aberrant RAS-RTK and hedgehog pathways, positioning them to be utilized for initial in vitro therapeutic screening as has been described in humans [42].

Limitations within this work include that the findings on DNA and RNA analysis were not validated with the use of a secondary testing methodology, such as polymerase chain reaction or immunohistochemistry. Yet the repeatability of the findings across all cell lines and parent tumors, as well as the comparison with historical controls, helps to act as internal validation of our results. Further, when comparing our data solely to historical results (parent tumor to hg and cell lines to hg) without inclusion of any of the historical data set, there is an introduction of batch effect which must be considered. Last, the hierarchical clustering of transcriptomic data was restricted to DEGs pre-selected between tumor and healthy gingiva samples, which introduces a selection bias that favors the discrimination of these two groups and may artificially position the cell lines in an intermediate location between healthy tissue and parent tumors. Reanalysis using all shared expressed genes across datasets would have provided a more unbiased view of global transcriptional relationships and should be considered for future work. Despite this, we were able to show maintenance of similar genetic aberrations within our cell lines and primary tumors compared to controls that were seen within historical publications [9].

In summary, we have generated 3 new canine CAA cell lines that harbor the most common mutations seen within the primary tumors and can act as a valuable tool in preclinical studies for treating ameloblastoma.

## Supporting information

S1 Fig Copy Number Alterations of the Primary Tumors (parent tumors) and the corresponding Established Cell Lines. There was no chromosol loss or gain in two of the parent tumors (A,E). The parent tumor to CAA 2 had a subclone (approximately 20% of cells) with a chromosome 27 deletion (B). CAA-2 had a subclone (70% of cells) with chromosome gain (D). CAA 3 also had a subclone (81% of cells) with chromosome gain (F).
(JPG)

S2 Fig Mutation Co-occurrence. Although many mutations occurred together with none being mutually exclusive, there was no significant correlation between mutations.
(JPG)

S1 Table Top Mutations among the cell lines and Primary Tumors (parent tumors). The top 12 mutations, including the chromosome location and allele shift are listed.
(TSV)
