# Supplementary figures and images for: Establishment and characterization of three canine ameloblastoma cell lines with genomic and transcriptomic profiles
Source: PLoS One. 2026 Jul 21;21(7):e0353197. doi: 10.1371/journal.pone.0353197 (PMC13387547; doi:10.1371/journal.pone.0353197)

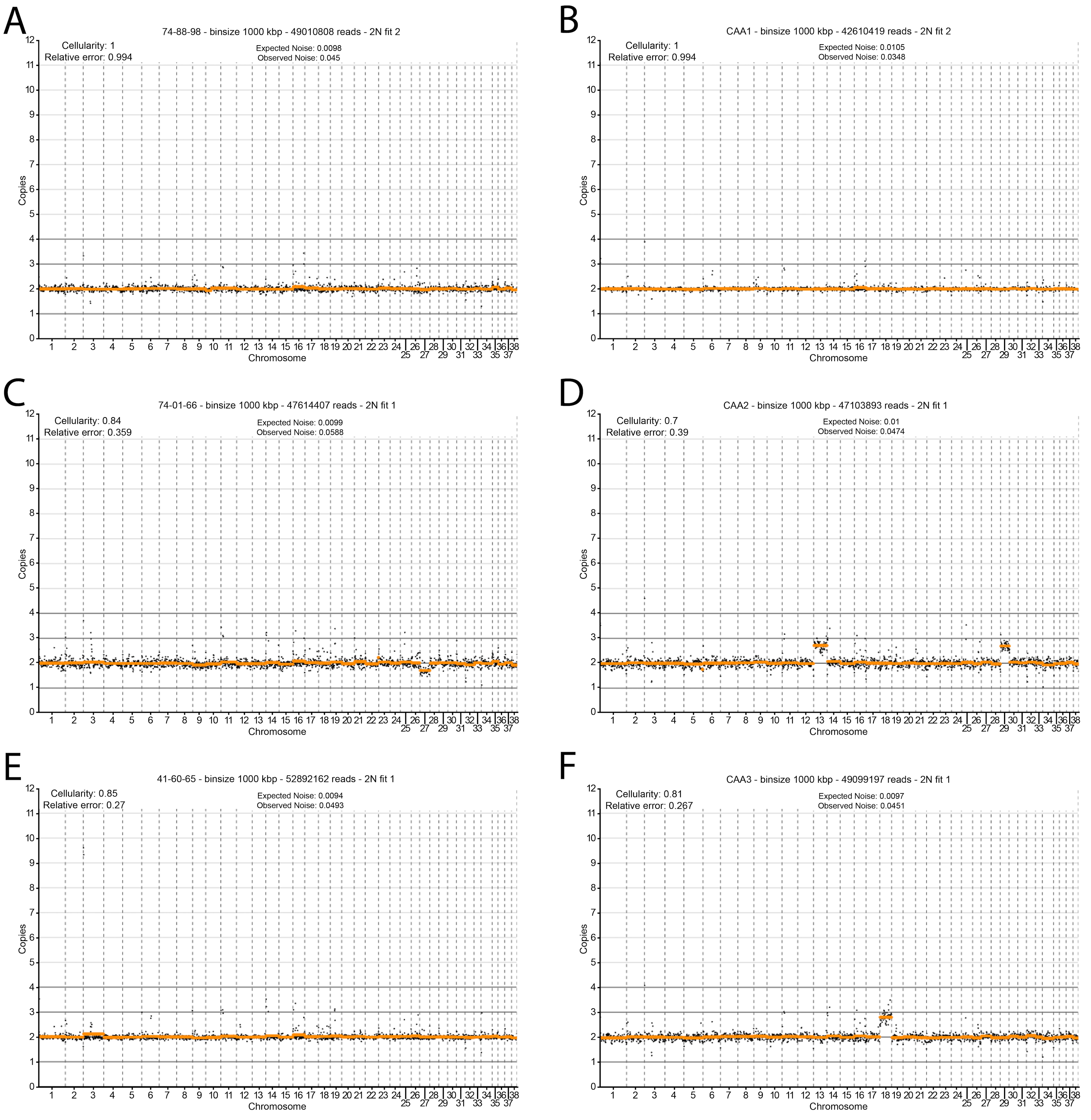

Supplement: S1 Fig — There was no chromosol loss or gain in two of the parent tumors (A,E). The parent tumor to CAA 2 had a subclone (approximately 20% of cells) with a chromosome 27 deletion (B). CAA-2 had a subclone (70% of cells) with chromosome gain (D). CAA 3 also had a subclone (81% of cells) with chromosome gain (F). (JPG) [file pone.0353197.s001.jpg]

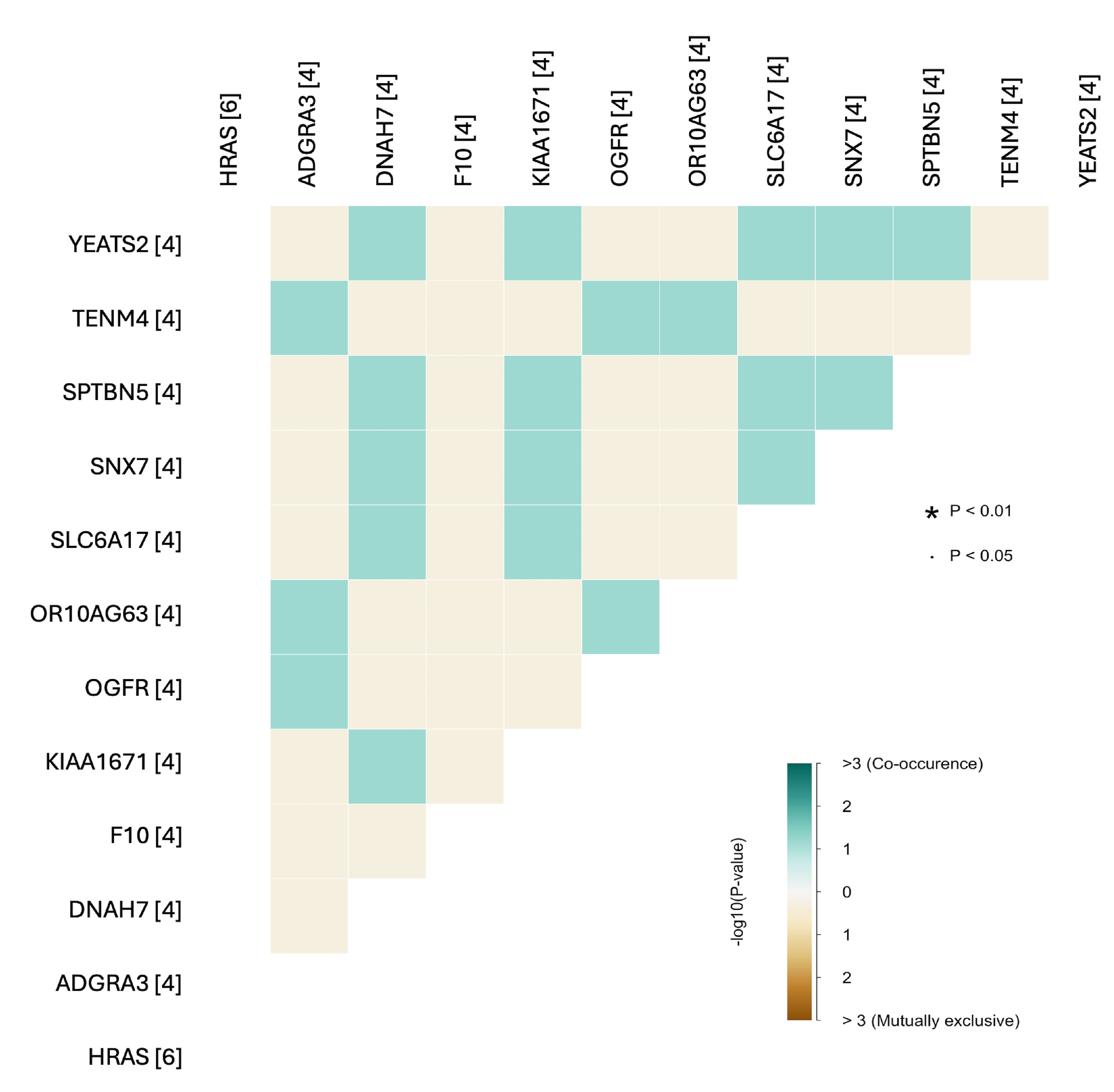

Supplement: S2 Fig — Although many mutations occurred together with none being mutually exclusive, there was no significant correlation between mutations. (JPG) [file pone.0353197.s002.jpg]
